# RETRACTION: Effect of Concomitant Tricuspid Annuloplasty on Postoperative Wound Complications in Heart Failure Patients Undergoing Mitral Valve Repair

**DOI:** 10.1111/iwj.70408

**Published:** 2025-03-21

## Abstract

**RETRACTION:** HouY. , GuoL. , LiY. , and CaiJ. , “Effect of Concomitant Tricuspid Annuloplasty on Postoperative Wound Complications in Heart Failure Patients Undergoing Mitral Valve Repair,” International Wound Journal 21, no. 5 (2024): e14835, 10.1111/iwj.14835.38786547 PMC11120391

The above article, published online on 24 May 2024, in Wiley Online Library (http://onlinelibrary.wiley.com/), has been retracted by agreement between the journal Editor in Chief, Professor Keith Harding; and John Wiley & Sons Ltd. Following an investigation by the publisher, all parties have concluded that this article was accepted solely on the basis of a compromised peer review process. The editors have therefore decided to retract the article. The authors did not respond to our notice regarding the retraction.

**RETRACTION:** Y. Hou , L. Guo , Y. Li , and J. Cai , “Effect of Concomitant Tricuspid Annuloplasty on Postoperative Wound Complications in Heart Failure Patients Undergoing Mitral Valve Repair,” International Wound Journal 21, no. 5 (2024): e14835, 10.1111/iwj.14835.38786547 PMC11120391

The above article, published online on 24 May 2024, in Wiley Online Library (http://onlinelibrary.wiley.com/), has been retracted by agreement between the journal Editor in Chief, Professor Keith Harding; and John Wiley & Sons Ltd. Following an investigation by the publisher, all parties have concluded that this article was accepted solely on the basis of a compromised peer review process. The editors have therefore decided to retract the article. The authors did not respond to our notice regarding the retraction.

